# Editorial: Pipeline inspection robots

**DOI:** 10.3389/frobt.2024.1497809

**Published:** 2024-11-05

**Authors:** Lyudmila Mihaylova, Atsushi Kakogawa, Shugen Ma, Hyouk Ryeol Choi, Taro Nakamura

**Affiliations:** ^1^ School of Electrical and Electronic Engineering, University of Sheffield, Sheffield, United Kingdom; ^2^ Graduate School of Science and Engineering, Ritsumeikan University, Kyoto, Japan; ^3^ Robotics and Autonomous Systems Thrust, Systems Hub, The Hong Kong University of Science and Technology (Guangzhou), Guangzhou, China; ^4^ School of Mechanical Engineering, Sungkyunkwan University, Jongno-gu, Republic of Korea; ^5^ Department of Precision Mechanical Engineering, Chuo University, Hachioji, Japan

**Keywords:** robotics, artificial intelligence simultaneous robot localisation and mapping (SLAM), robot control, pipe networks, inspection, maintenance

## 1 Introduction

Recent years have witnessed an incredible progress in Artificial Intelligence (AI), Machine Learning, Robotics and Digital Technologies. The Research Topic: “*Pipeline Inspection Robots*” demonstrates the power of AI to introduce autonomy is difficult underground environments, not so friendly for human operations.

This Research Topic identifies challenges and proposes potential solutions to autonomous robot tasks in pipe networks. Some of the challenges are due to a number of factors: the infrastructure (e.g., winding pipes, curved or branched), the sensors and intelligent sensing methods, robot resilience to sub-terrain environments and GPS-denied communications. This Research Topic introduces the state-of-the-art research on pipeline inspection robots and contains contribution articles showing the potential of autonomous robots to revolutionise work and empower maintenance teams in their difficult tasks. This Research Topic also brings together papers from very different perspectives: autonomous control of in-pipe inspection robots, robot localization, environmental adaptation mechanisms, and safety in collaborative robotics.

We called for the participation of researchers and engineers working in this field and we collected the contributions on the topics described in the next section. Four papers were accepted as a result of the call for manuscripts.

## 2 Overview of the contents of the research topic

Article (Nauert and Kampmann) identifies key requirements to autonomous underwater robots and major challenges which need to be addressed in development of new technologies. High level of autonomy constitutes the main challenge. The paper gives an overview of works for inspection, including inspection with visual, acoustic and electromagnetic methods. Different inspection and maintenance tasks performed by industry are discussed in connection with the technology and the current state-of-the-art of underwater robots is presented. This work focuses on maintenance and inspection from the outside of the underwater pipe. The inspection and maintenance tasks, required by the industry and the currently employed tooling, are described and the current state of underwater robots was presented.

In (Kakogawa and Ma) an in-pipe crawler robot is developed. It has a modular structure and can automatically shift its body shape. The robot can shift its body shape to a parallelogram and adapts to obstacles and environmental conditions thanks to the crawlers. The influence of the robot roll angle, torques, parameters and external factors are studied in detail. The impact of the initial robot resistance is also thoroughly studied. The advantages of the designed mechanism are demonstrated over real experiments.

Pipe networks challenge vision-based navigation systems since there are no well pronounced features in images, such as corners, lines and repeated texture. To address this challenge, Edwards et al. develop a method for robot localisation in pipe networks that is able to deal with the sparse image features. The importance of approximate localization methods operating in one degree-of-freedom environment along the pipe length that are robust to environmental changes is discussed with respect to accurate odometry methods, operating in six degrees of freedom, but requiring well visible image features.

Nguyen et al. introduce a miniaturised mobile robot with efficient control for pipe inspection. The aim is to fully automate the inspection of 75-mm-diameter sewers without any visual aid or power-intensive image processing tools. The wheel-legs mechanism allow the robot to move through uneven terrains. The robot implements an interesting idea about a high-level control and low-level control. Low-level control is primarily aimed at achieving stability, moving to and along the center of pipes. High-level control is used to determine the local robot state, and for during decision making.

However, the common points among them are how we develop the high performance robot within the extremely large space limitation and the harsh environment. This requires how to maximize the performance under limited conditions, which is very useful information not only for pipe inspection robots but also for many other robotics research and development efforts in terms of robot size and weight reduction, simplification, cost reduction, etc. We hope that the results of this Research Topic will be helpful to readers in a wide range of fields and lead to further development. Computational aspects and efficiency are essential since these robots operate under constraints, including from energy perspectives.

## 3 Conclusion

The creation of miniature robots, equipped with computational and communication resources requires further attention. This issue presents current developments from this area.

